# Group C *Neisseria meningitidis* as a Cause of Septic Arthritis in a Native Shoulder Joint: A Case Report

**DOI:** 10.1155/2011/862487

**Published:** 2011-10-19

**Authors:** Amy J. Garner, Freda Sundram, Kathryn Harris

**Affiliations:** ^1^Barnet General Hospital, Wellhouse Lane, Herts, Barnet EN5 3DJ, UK; ^2^Great Ormond Street Hospital, Great Ormond Street, London WC1N 3JH, UK

## Abstract

Septic arthritis is an Orthopaedic emergency, threatening the joint within hours of onset. Up to 10% of cases of meningococcaemia have an associated septic arthritis. The aetiology of acute meningococcaemia in a variety of clinical syndromes is well documented in the literature. The pathogen *Neisseria meningitidis* can cause both primary and secondary manifestations of disseminated infection. Broad-range 16S rDNA PCR is a relatively new technique, useful in identifying aetiological agents in septic patients with negative blood cultures. Here, we describe the rare clinical scenario of a 76-year-old woman with primary meningococcal septic arthritis of a native shoulder joint without associated meningococcal bloodstream infection. We discuss the role of 16s rDNA Polymerase Chain Reaction (PCR) in the identification of the infectious agent, *Neisseria meningitidis*, and the role of this technique in guiding subsequent management.

## 1. Introduction

Acute septic arthritis refers to pyogenic inflammation of a joint space as a result of bacterial infection. Bacteria spread to the joint most often through a haematogenous route. Direct invasion, for example, during trauma, or contiguous spread from periarticular tissue harbouring infection is also well described. 80% of cases of septic arthritis result from infection with Gram-positive aerobes, with *Staphylococcus aureus* being the most common etiological agent [1]. Septic arthritis of the acromiocavicular joint is relatively rare. Here we report a case of septic arthritis of the native shoulder joint with *Neisseria meningitidis*—a pathogen rarely associated with this condition. We report our challenge to identify the responsible organism and discuss the innovative techniques used in its detection.

## 2. Case Report

A 76-year-old female presented to the emergency department with a three-day history of pain in the right shoulder associated with fever and profuse sweating. The patient initially presented to another hospital, two days earlier, with the same complaint. On that occasion she was treated with an oral course of erythromycin for a presumed skin infection. There was no history of trauma. The shoulder had never been invaded, with a therapeutic injection, for example. The patient did not complain of headache, neck stiffness, or photophobia. No other joints were affected. 

The patient's past medical history included hypertension, hypercholesterolaemia, dilated cardiomyopathy, gastroesophageal reflux disease, recurrent urinary tract infections, and gallstones. An uncomplicated elective total hip replacement had been performed five years earlier for osteoarthritis. Current medications included Irbesartan; Bisoprolol; Aspirin; Furosemide; Atorvastatin; Mebeverine; Lansoprazole; Domperidone; Quinine and a prophylactic dose of Nitrofurantoin in addition to oral Erythromycin, taken for two days at 500 mg QDS.

On examination in our hospital, the patient appeared flushed, lethargic, and complained of considerable pain in the right, dominant shoulder. Her temperature was 37.6°C. The blood pressure was 91/53 mmHg without a tachycardia. There was no photophobia or rash. The patient had a full range of movement in the neck. The right shoulder appeared oedematous, warm, and erythematous. Active shoulder movement was minimal, limited by pain. Passively, the shoulder could be moved no greater than 20 degrees in any plane. A 5 mm discrete erythematous papular soft tissue lesion was noted on the lateral border of the arm 15 cm distal to the glenohumeral joint. 

**Figure 1:**
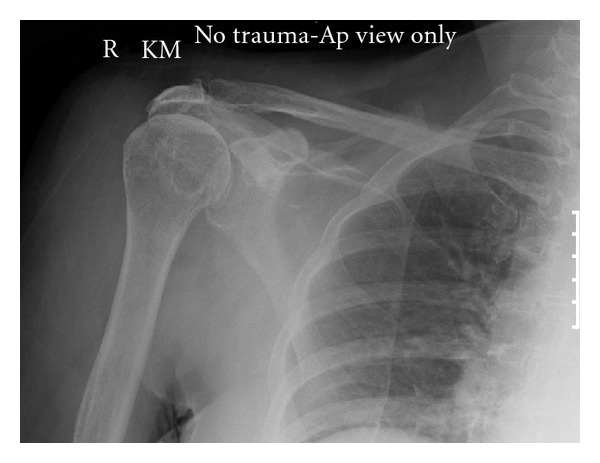
Anterior-posterior radiograph of the right shoulder at admission.

X-ray of the shoulder joint (Figure 1) revealed significant osteoarthritis and reduced joint space. Prior to starting intravenous antibiotic therapy, approximately 60 mL of purulent material was aspirated from the right shoulder revealing a high number of pus cells. No organisms were seen on microscopy and Gram stain. Intravenous Benzylpenicillin and Flucloxacillin were started immediately after aspiration of the joint and drawing of peripheral blood cultures. The peripheral white blood cell count was 14.9 × 10^9^/*μ*L (neutrophils 12.5 × 10^9^/*μ*L), and the C-reactive protein (CRP) was 262 mg/L. 

The patient did not display any evidence of meningism and the aspirate was inconclusive, therefore, no clinical need to perform cerebrospinal fluid (CSF) analysis presented itself and was therefore not performed.

**Figure 2:**
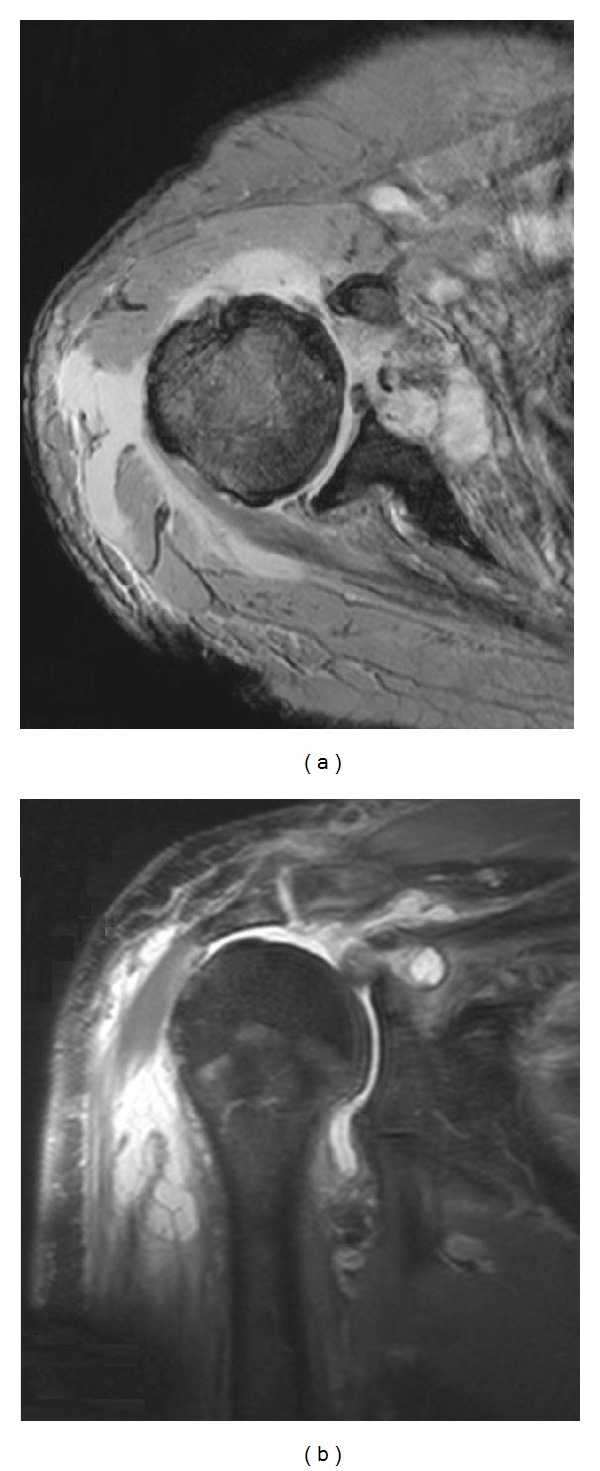
T2 Weighted MRI Images of the right shoulder taken at 22 hours after presentation, showing early osteomyelitis, bone marrow oedema, bursal collection, and high signal consistent with septic arthritis.

An MRI scan of the shoulder was performed (Figures 2(a) and 2(b)) followed by an open washout of the joint. During the procedure a significant volume of purulent material was removed from the joint capsule and sent for microscopy and culture. Glenohumeral arthritis was noted intraoperatively. An anterior drain was left in situ. The patient remained septic for 48 hours with recurrent fevers and a persistently high white cell count (12.1 × 10^9^/*μ*L). A second washout procedure was therefore performed at 72 hours after the first, during which, the relatively dry capsule was swabbed for microscopy and culture. 

**Figure 3:**
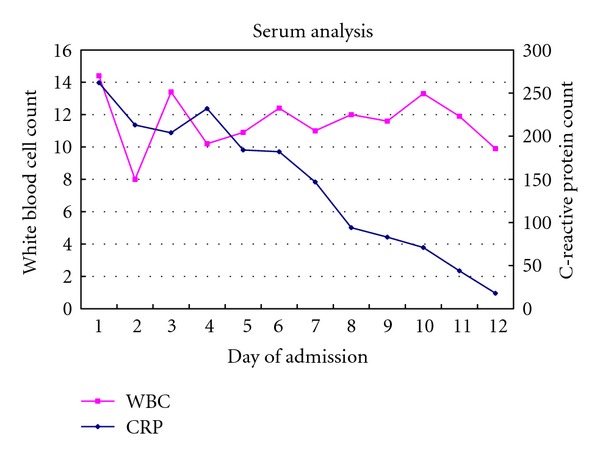
Serum analysis of white cell count and C-reactive protein levels during admission. Antibiotic (ABx) switch at day 10 from Benzylpenicillin and Flucloxacillin to Teicoplanin and Fusidic Acid for presumed MSSA.

The patient responded well clinically over the subsequent week. The CRP fell consistently though the white cell remained high (Figure 3). Intravenous Benzylpenicillin and Flucloxacillin were continued via a Peripherally Inserted Central Catheter (PICC) line on an outpatient basis. 

Despite the clinical picture of septic arthritis and the purulent nature of the aspirate, neither the initial joint aspirate nor peripheral blood cultures revealed any organisms on culture. The joint aspirate was sent for a broad-range 16S rDNA PCR, and the patient was started on a six-week course of intravenous Teicoplanin and oral Fusidic acid was started for presumptive meticillin-sensitive *Staphylococcus aureus* (MSSA) infection. 

Two weeks later, the 16S rDNA PCR returned a result of *Neisseria meningitidis *from the original aspirate. The sample was cross-referenced with the Meningococcal Reference Laboratory and was confirmed to be a Group C *N. meningitidis*. The Reference Laboratory is in Manchester, and they play a useful role in guiding antibiotic therapy as well as Public Health decisions regarding prophylaxis. A PCR technique is performed in order to identify various strains of meningococci from blood, CSF, and tissue samples. 

The antibiotic therapy was switched accordingly to intravenous Ceftriaxone and oral Rifampicin. The patient failed to tolerate the Rifampicin and continued on Ceftriaxone as monotherapy for 12 weeks. The department of Public Health was informed, and close contacts were given Rifampicin and immunized against *N. meningitidis* serogroup C.

## 3. Discussion

Septic arthritis is usually caused by staphylococcal and streptococcal species. Unusual organisms, such as meningococci, are rare pathogens and mainly affect people with immunodeficiencies without the clinical syndromes associated with meningococcaemia [2].

The clinical manifestations of meningococcal disease can be quite varied, ranging from transient fever and bacteraemia to fulminant disease with death ensuing within hours of the onset of clinical symptoms. 

Approximately 2% to 10% of cases of acute meningococcal infection are associated with some form of articular dysfunction [3]. These occur through a variety of mechanisms, including direct bacterial seeding via the bloodstream and immune complex deposition within the joint capsule. In some cases, the joint can be completely destroyed within hours of developing infection. 

Meningococcal serogroups A, B, and C release lipooligosaccharide from their membrane surfaces, and this is now considered to be the principal factor associated with high endotoxin levels in meningococcal sepsis [4]. 

Primary meningococcal arthritis (PMA) represents a rare form of meningococcal disease and is not well described outside the realm of associated meningococcaemia. Giamerellis-Bourbolis et al. reported 34 cases of PMA in the literature from 1980 to 2002 [5]. Pre existing joint disease is a major risk factor and is found in 47% of patients diagnosed with septic arthritis [6]. 

The presentation of PMA can be very similar to septic arthritis caused by *Neisseria gonorrhoeae*, as part of disseminated gonococcal infection. Both bacteria can cause a monoarthritis and a rash. Direct bacterial invasion of the synovium via the bloodstream is the proposed pathogenesis of PMA, with approximately 40% of patients having positive blood cultures [7]. It is imperative to isolate the organism from blood and joint fluid, in order to initiate and continue appropriate treatment.

In our case, all tissue and blood cultures remained negative, perhaps due to ongoing intravenous therapy with Benzylpenicillin. Molecular techniques are increasingly employed in order to improve the diagnosis of bacterial infection in specimens, which are deemed culture negative either by prior empirical antibiotic treatment or due to the presence of fastidious organisms. Reports in the literature describe rates of isolation of between 25 and 50% of live or viable organisms by routine culture in both these culture-negative groups. Hence, detection of a fastidious or atypical pathogen is essential to guide the choice of prolonged antibiotic therapy in patients with deep-seated infections.

The 16S rRNA gene (16S rDNA) codes for a component of the prokaryotic ribosome, and it contains DNA sequences that are highly conserved between different species of bacteria. Broad-range 16S rDNA PCR has been successfully applied in numerous clinical settings, including the diagnosis of septic arthritis in children [8]. PCR is performed using primers targeting conserved regions of the bacterial 16S rDNA. The resulting amplicon, which contains variable regions of DNA, is sequenced and compared with other 16S rDNA sequences in the Genbank database using BLAST [9] or by comparison to an in-house database.

The sensitivity of broad-range bacterial PCR is generally lower than real-time PCR for a single bacterial target because of the need to avoid detection of ubiquitous bacterial DNA contamination of reagents, which is well documented [10].

Use of this technique can detect and identify DNA from a wide range of organisms in material from normally sterile sites and increases the diagnosis of bacterial infection from culture-negative clinical specimens when used alongside routine culture techniques. However, there are several limitations to its use, including lack of sensitivity, since culture is the most sensitive technique for detecting viable organisms and lack of information on the antibiotic sensitivities of the organism, which presents a significant challenge in the current age of increasing antibiotic resistance. However, broad-range 16S rDNA is a useful technique in the clinical microbiology laboratory that can improve the diagnosis of bacterial infections from culture-negative samples. Though, ultimately, the significance of the etiological agent identified should be carefully interpreted within the context of the clinical setting.

## 4. Limitations

This case highlights several management learning points. The literature dictates that any hot swollen tender joint in an unwell patient is septic arthritis until proven otherwise. The diagnosis of a skin infection, and subsequent treatment, by another hospital, with erythromycin prior to aspiration of the joint, almost certainly impeded subsequent management and isolation of the infecting organism in this case. 

Further, arthroscopic washout in septic arthritis is regarded by many as superior to open washout, repeated where necessary. However, in this case the patient's clinical condition mandated urgent transfer to theatre at a time when no arthroscopic equipment was available. The surgeon opted to proceed in an open procedure with the view that open washout would be of greater benefit than waiting for arthroscopic equipment to become available. The subsequent procedure was conducted through the same incision and was therefore also an open washout. Theatre policies within the hospital have now been amended so that an emergency arthroscopic set is now available at all times should a similar case arise in the future.

##  Conflict of Interests

The authors have no conflict of interests.
